# GLP-1 AND CIRRHOSIS: EFFECTS ON MORTALITY AND LIVER-RELATED COMPLICATIONS

**DOI:** 10.1590/S0004-2803.24612025-130

**Published:** 2026-06-05

**Authors:** Chidera N ONWUZO, Odusanya Kikunlore ELIJAH, FNU ALVINA, Rashid Abdel RAZEQ, KOJO-FRIMPONG B. AWUAH, Somtochukwu ONWUZO

**Affiliations:** 1 SUNY Upstate Medical University, Internal Medicine Department, Resident Physician, Syracuse, NY, USA.; 2 Allegheny General Health, Gastroenterology Department, Fellow Physician, Pittsburgh, PA, USA.; 3 Cleveland Clinic Fairview Hospital, Internal Medicine Department, Resident Physician, Cleveland, OH, USA.; 4 Olabisi Onabanjo University Teaching Hospital, Hematology Oncology Department, Resident Physician, Ogun State, Nigeria.

**Keywords:** GLP-1 analogs, cirrhosis, hepatorenal syndrome, hepatic encephalopathy, mortality, análogos de GLP-1, cirrose, síndrome hepatorrenal, encefalopatia hepática, mortalidade

## Abstract

**Background:**

Cirrhosis is characterized by complications, including hepatic encephalopathy (HE), hepatorenal syndrome (HRS), and acute variceal bleeding, Glucagon-like peptide-1 (GLP-1) analogs, have shown potential benefits in reducing liver inflammation and lowering cirrhosis-related complications. This study aims to provide insights into the effect of GLP-1 therapy on mortality and clinical outcomes in patients with cirrhosis.

**Methods:**

We conducted a retrospective analysis using TriNetX research network. The cohort comprised patients ≥18 years old with a diagnosis of cirrhosis. Patients who were started on GLP-1 analogs after the diagnosis of cirrhosis were identified. Primary outcomes included all-cause mortality, HE, HRS, and portal hypertensive bleeding. Variables such as age, sex, race, comorbidities (e.g., diabetes, obesity, chronic kidney disease, hypertension), and lifestyle factors underwent 1:1 propensity matching to reduce confounding. Cox proportional hazards regression analysis was utilized to analyze the matched cohorts, and hazard ratios (HR) with 95% confidence intervals.

**Results:**

GLP-1 analog use was significantly associated with a reduced risk of several cirrhosis-related complications. Patients treated with GLP-1 analogs exhibited a significantly lower risk of all-cause mortality (HR 0.374, 95%CI 0.359-0.392), HE (HR 0.900, 95%CI 0.843-0.959), and HRS (HR 0.554, 95%CI 0.496-0.619). Additionally, there was reduced rates of portal hypertensive bleeding among GLP-1 users (HR 0.487, 95%CI 0.444-0.533).

**Conclusion:**

This study demonstrates that GLP-1 analog use is associated with a significantly lower risk of all-cause mortality and cirrhosis-related complications. Furthermore, the reduced rate of portal hypertensive bleeding might correlate with subsequent decrease in hospitalizations.

## HIGHLIGHTS

• TriNetX cohort: adults with cirrhosis initiating GLP-1 after diagnosis.

• 1:1 propensity-score matching; Cox models for mortality and complications.

• GLP-1 use linked to lower all-cause mortality (HR 0.37; 95% CI 0.36-0.39).

• Lower hepatorenal syndrome risk (HR 0.55) and portal hypertensive bleeding (HR 0.49).

• Modest reduction in hepatic encephalopathy risk (HR 0.90).

## RESUMO

**Contexto:**

A cirrose é caracterizada por complicações, incluindo encefalopatia hepática (EH), síndrome hepatorrenal (SHR) e sangramento varicoso agudo. Os análogos do peptídeo-1 semelhante ao glucagon (GLP-1) têm demonstrado benefícios potenciais na redução da inflamação hepática e na diminuição de complicações relacionadas à cirrose. Este estudo visa fornecer insights sobre o efeito da terapia com GLP-1 na mortalidade e nos desfechos clínicos em pacientes com cirrose.

**Métodos:**

Realizamos uma análise retrospectiva utilizando a rede de pesquisa TriNetX. A coorte incluiu pacientes com ≥18 anos com diagnóstico de cirrose. Foram identificados pacientes que iniciaram análogos de GLP-1 após o diagnóstico de cirrose. Os desfechos primários incluíram mortalidade por todas as causas, EH, SHR e sangramento por hipertensão portal. Variáveis como idade, sexo, raça, comorbidades (por exemplo, diabetes, obesidade, doença renal crônica, hipertensão) e fatores de estilo de vida foram submetidas a pareamento por escore de propensão 1:1 para reduzir confundimento. A regressão de riscos proporcionais de Cox foi utilizada para analisar as coortes pareadas, e foram estimadas razões de risco (HR) com intervalos de confiança de 95%.

**Resultados:**

O uso de análogos de GLP-1 esteve significativamente associado a menor risco de várias complicações relacionadas à cirrose. Pacientes tratados com análogos de GLP-1 apresentaram risco significativamente menor de mortalidade por todas as causas (HR 0,374; IC95%: 0,359-0,392), EH (HR 0,900; IC95%: 0,843-0,959) e SHR (HR 0,554; IC95%: 0,496-0,619). Além disso, houve menor taxa de sangramento por hipertensão portal entre usuários de GLP-1 (HR 0,487; IC95%: 0,444-0,533).

**Conclusão:**

Este estudo demonstra que o uso de análogos de GLP-1 está associado a um risco significativamente menor de mortalidade por todas as causas e de complicações relacionadas à cirrose. Além disso, a redução da taxa de sangramento por hipertensão portal pode correlacionar-se com diminuição subsequente de hospitalizações.

## INTRODUCTION

Cirrhosis develops after a long period of inflammation that results in replacement of the healthy liver parenchyma with fibrotic tissue and regenerative nodules, leading to portal hypertension^1^. It represents the end-stage of chronic liver disease and is a leading global cause of morbidity and mortality, with over 40,000 deaths each year in the United States and over 1.16 million deaths annually worldwide^2^. Among its many etiologies, non-alcoholic fatty liver disease (NAFLD), a metabolic liver condition that ranges from simple steatosis to nonalcoholic steatohepatitis (NASH), fibrosis, and cirrhosis has emerged as a major driver, especially given its strong association with type 2 diabetes mellitus (T2DM) and obesity^3^. As these metabolic disorders have risen globally, so has the burden of NAFLD-related cirrhosis, making it one of the most common causes of liver-related deaths^4^.

Approximately 70% of patients with cirrhosis exhibit glucose intolerance and about 30% have T2DM, resulting from impaired hepatic insulin clearance, portosystemic shunting, insulin resistance, and pancreatic β-cell dysfunction^5^ ^,^ ^6^. T2DM not only accelerates hepatic fibrogenesis but is also a strong predictor of cirrhosis-related complications, including hepatic encephalopathy, ascites, variceal bleeding, hepatorenal syndrome, and hepatocellular carcinoma (HCC)^6^. While glycemic control is a cornerstone of T2DM management, its role in cirrhosis is complex and controversial due to altered drug metabolism and increased risk of adverse events.

Glucagon-like peptide-1 receptor agonists (GLP-1RAs) approved since 2005 for T2DM, have demonstrated broad metabolic benefits; including glucose-dependent insulin secretion, glucagon suppression, delayed gastric emptying, appetite regulation, and weight loss^4^ ^,^ ^7^. Emerging evidence also points to their hepatic benefits, including reduced liver fat content, improvement in steatohepatitis, and favorable changes in hepatic lipid oxidation and inflammation in both preclinical and human studies^8^ ^,^ ^9^. Notably, GLP-1RAs have shown efficacy in reversing NASH and improving histologic liver outcomes in patients without cirrhosis^10^.

However, despite these promising metabolic and hepatoprotective effects, the safety and effectiveness of GLP-1RAs in patients with established cirrhosis remains largely unexplored^10^. Given the unique pathophysiology and high risk of adverse outcomes in cirrhotic patients, particularly those with decompensation, there remains a critical need to identify antidiabetic therapies that are both safe and potentially disease-modifying in this population.

This study aims to address this knowledge gap by evaluating the association between GLP-1RA use and clinical outcomes in patients with cirrhosis, including liver-related complications and all-cause mortality. By addressing this understudied yet clinically important population, we seek to elucidate the potential of GLP-1 RAs to alter the disease trajectory even in advanced liver disease.

## METHODS

### Study design, setting and participants

We performed a retrospective cohort study of adults with cirrhosis utilizing patient data from the TrinetX US Collaborative research network, a large multisystem database with over 130 million patients. This retrospective cohort study included adults diagnosed with cirrhosis and using GLP1 agonists matched to those of the same age group with cirrhosis but not on GLP1 agonist. Patients with cirrhosis were identified via ICD-10 codes. Propensity score matching was then performed to ensure balanced demographic and clinical characteristics between the GLP1 agonists and non-GLP1 agonist cohorts.

The diagnosis of cirrhosis was determined using the International Classification of Diseases, Tenth Revision, Clinical Modification (ICD-10-CM). For data originally coded in ICD-9-CM, the study utilized TriNetX’s mapping to convert it to ICD-10-CM, using general equivalence mappings and custom algorithms to ensure accuracy. RxNorm was used to identify patients on GLP1 agonist and patients who were not. The patient population was segregated into two groups: cirrhosis patients on GLP1 agonists and cirrhosis patients not on GLP1 agonist.

### Data source and de-identification

We extracted data from the TriNetX global collaborative network, a federated research platform aggregating electronic health records from a diverse range of healthcare organizations across 30 countries worldwide. TriNetX adheres to the Health Insurance Portability and Accountability Act (HIPAA) standards to ensure that all patient-level and aggregated data are de-identified, following the guidelines of the HIPAA Privacy Rule, particularly Section §164.514[a]. The de-identification process is validated by a qualified expert as required by Section §164.514[b] of the HIPAA Privacy Rule, negating the need for a prior waiver from the Western Institutional Review Board (IRB). Over 70 healthcare organisations who contributed de-identified data to TriNetX included academic university hospitals, community hospitals, and specialty physician services covering inpatient and outpatient care.

### Propensity score matching

To address baseline imbalances, we applied 1:1 greedy nearest-neighbour propensity score matching with a caliper of 0.1. Covariates included age, sex, race, ethnicity, lifestyle factors (e.g., tobacco use, alcohol dependence], and comorbidities (e.g., diabetes, obesity, chronic kidney disease, hypertension, hyperlipidemia). Propensity scores were estimated using logistic regression, and matching was performed without replacement, meaning each control patient could be matched to only one treated patient, ensuring unique 1:1 pairs. Following matching, both groups comprised 22,889 patients with balanced baseline characteristics.

### Outcomes and statistical analysis

The primary outcomes were all-cause mortality, hepatic encephalopathy, hepatorenal syndrome, and portal hypertensive bleeding. Post-match balance was evaluated using standardized mean differences (SMD), with SMD <0.1 indicating negligible imbalance. For all-cause mortality, survival analysis was conducted using a Cox proportional hazards regression model to estimate hazard ratios (HRs) with 95% confidence intervals. Hazard ratios with 95% confidence intervals were also calculated for each of the other outcomes, treating the matched pairs as independent observations given the large sample size and verifying that conditional logistic regression produced consistent estimates. Effect sizes and confidence intervals were visually summarized in forest plots. All tests were two-tailed, with *P*<0.05 considered statistically significant. Analyses were performed with the TriNetX platform and independently verified in R version 4.2.2.

## RESULTS

**FIGURE 1:**
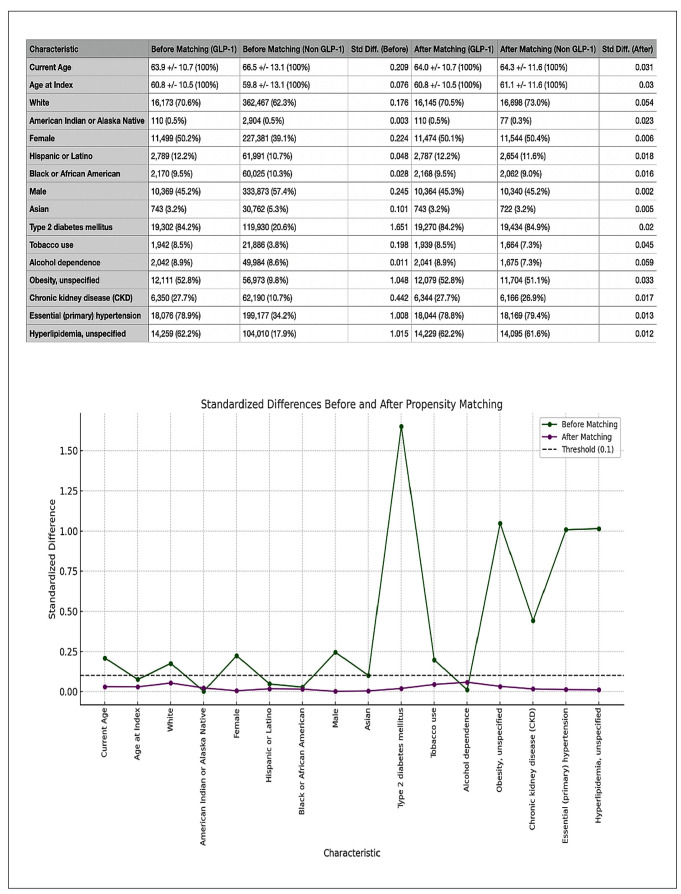
Comparison of demographics and comorbidities of GLP-1 analog users and Non-GLP-1 analog users before and after propensity score matching.

We identified patients with cirrhosis confirmed by ICD-10 codes, after applying exclusion criteria, including 22,921 in cohort 1 and 607,737 in cohort 2. After PS-matching, the glp cohort (n=22,889) had mean age of 60.8±10.5 years, also included 10,364 men (45.3%), 11474 (50.1%) women, 16,145 Whites (70.5%), 110 American Indian or Alaska native (0.5%), 2,168 Blacks or African American (9.5%), 743 Asians (3.2%) and 2,787 Hispanic/Latino (12.2%) patients. The non GLP cohort (n=22,889) had mean age of 61.1±11.6 years and included 10,340 men (45.2%), 11,544 (50.4%) women, 16,698 Whites (73%), 77 American Indian or Alaska native (0.3%), 722 Asians (3.2%) and 2654 Hispanic/Latino patients (11.6%). Figure 1 outlines the preceding patient diagnoses associated with the cirrhosis burden.

Significant differences were also noted in the prevalence of several comorbidities, including type 2 diabetes mellitus, chronic kidney disease, obesity, essential (primary) hypertension, and hyperlipidemia. Differences were also noted in lifestyle factors, including tobacco use and alcohol dependence.

### Primary outcomes

After PS-matching, we found that the GLP1-RA therapy group had decreased all cause mortality risk (hazard ratio (HR) 0.374, 95%CI 0.359-0.392) compared to the non glp group.

**FIGURE 2:**
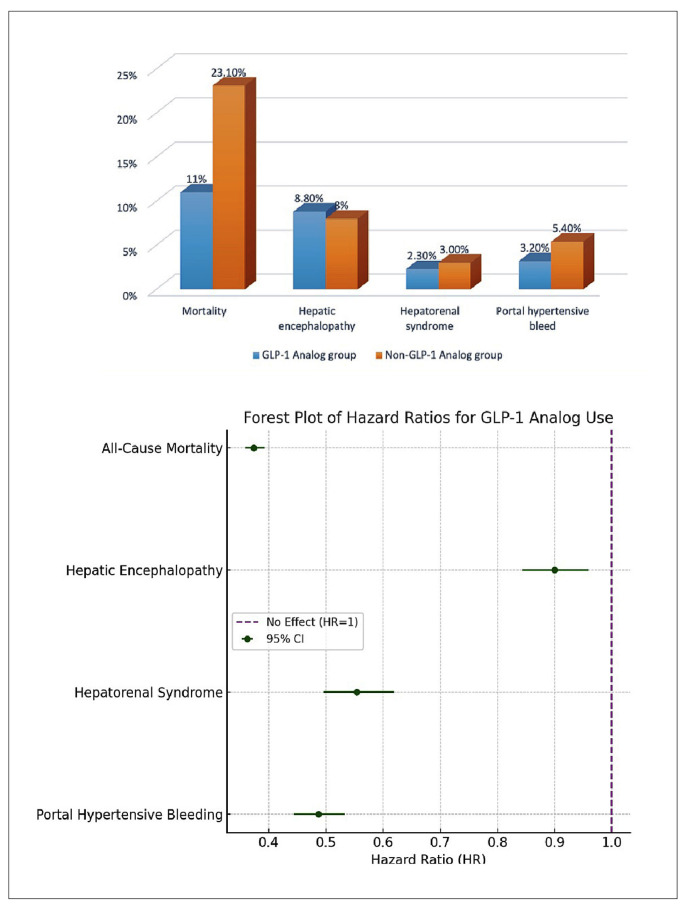
Bar chart showing complication rates & forest plot comparing hazard ratios for events in GLP analog group vs non-GLP analog group.

We also found that cirrhotic patients on GLP 1 agonists exhibited lower risk of hepatic encephalopathy (HR 0.900, 95%CI 0.843-0.959), hepatorenal syndrome (HR 0.554, 95%CI 0.496-0.619). Additionally, lower rates of portal hypertensive bleeding was seen among these users (HR 0.487, 95%CI 0.444-0.533) Figure 2.

## DISCUSSION

Our study revealed that GLP-1 receptor agonist (GLP-1RA) use was significantly associated with a reduced risk of several cirrhosis-related complications and decompensation events. Specifically, patients treated with GLP-1RAs demonstrated a significantly lower risk of all-cause mortality, hepatic encephalopathy, hepatorenal syndrome, and portal hypertensive bleeding. These findings suggest that GLP-1RA use may be beneficial in attenuating disease progression and improving survival in patients with cirrhosis, especially those with coexisting type 2 diabetes mellitus (T2DM).

Cirrhosis is associated with high morbidity and mortality, with annual mortality ranging from 1% in compensated stages to as high as 80% in decompensated disease^11^. Diabetes significantly worsens the prognosis in cirrhosis, accelerating progression to hepatic decompensation and liver-related death^12^ ^,^ ^13^. Glycemic control and cirrhosis management often have synergistic effects, and the use of antidiabetic agents with liver-specific benefits could offer dual therapeutic gains^14^ ^,^ ^15^.

Several mechanistic pathways may underlie the observed clinical benefits of GLP-1RAs in cirrhosis. These agents have been shown to reduce hepatic steatosis, lipotoxicity, and insulin resistance; mitigate oxidative and endoplasmic reticulum stress in hepatocytes; and modulate inflammation by reducing macrophage activity and improving mitochondrial function^6^ ^,^ ^16^ ^,^ ^17^. Additionally, they improve gut barrier function, reduce intestinal ammonia production, and alter gut microbiota which are all relevant to hepatic encephalopathy^6^ ^,^ ^10^. GLP-1RAs also promote neurogenesis and synaptic remodeling in the CNS, possibly contributing to improved neurocognitive function in cirrhotic patients with encephalopathy^6^.

GLP-1RAs induce satiety, slow gastric emptying, and reduce postprandial glucose and triglyceride excursions, contributing to better metabolic control^18^ ^-^ ^20^. Their cardioprotective and nephroprotective effects including reduced rates of major adverse cardiovascular events (MACEs), slowed decline in eGFR, and reduced albuminuria may also contribute to improved outcomes in cirrhosis, where cardiorenal complications are common^21^ ^-^ ^23^. These systemic benefits may explain the reduced incidence of hepatorenal syndrome observed in our study.

Our findings align with emerging literature supporting the hepatoprotective role of GLP-1RAs. Yen et al. reported significantly reduced risks of all-cause mortality (aHR 0.47), decompensated cirrhosis (aHR 0.7), hepatic encephalopathy (aHR 0.59), liver failure (aHR 0.54), and MACEs (aHR 0.6) in GLP-1RA users compared to nonusers^6^. Prolonged use (>179 days) was associated with even greater reductions. Similarly, Mohammed et al.^24^. demonstrated a 36% lower risk of hepatic decompensation in patients with MASLD cirrhosis and T2DM on GLP-1RA therapy. A randomized controlled trial of survodutide, a dual GLP-1/glucagon receptor agonist, showed it to be well-tolerated in both cirrhotic and non-cirrhotic patients, with meaningful reductions in liver fat, stiffness, and fibrosis biomarkers^16^.

A Swedish cohort study found that GLP-1RA initiation was associated with a 49% reduction in the 10-year risk of major adverse liver outcomes (MALO) in per-protocol analyses. However, intention-to-treat estimates were less precise, emphasizing the need for adherence and consistent follow-up^25^. Simon et al. also documented lower rates of cirrhosis-related complications including ascites, spontaneous bacterial peritonitis, hepatorenal syndrome, and variceal bleeding among GLP-1RA users compared to those on other antidiabetic drugs.

Preclinical data further support these findings. GLP-1RAs promote hepatic stellate cell quiescence, enhance lipid oxidation, suppress cholangiocyte apoptosis, and reduce inflammation, potentially influencing fibrosis and portal hypertension^26^ ^-^ ^28^. Though GLP-1 receptor expression in the liver remains debated, indirect effects via weight loss, glycemic control, and anti-inflammatory mechanisms may drive fibrosis regression over time^17^.

The hepatologic benefits of GLP-1RAs have been particularly evident in NAFLD and NASH populations^19^ ^,^ ^29^ ^,^ ^30^. Multiple randomized controlled trials have shown significant improvements in liver fat, inflammation, and resolution of steatohepatitis^31^ ^,^ ^32^. Our findings suggest these benefits may extend into the cirrhotic phase, a stage previously understudied in GLP-1RA research due to concerns over drug metabolism and tolerability in advanced liver disease.

Emerging data also suggest that GLP-1RAs may have behavioural and neuropsychiatric benefits relevant to cirrhosis care. Several studies have shown that GLP-1RA use is associated with reduced alcohol intake and cravings, likely via central reward pathway modulation^33^ ^-^ ^35^. Given the profound impact of alcohol abstinence on cirrhosis outcomes, this additional benefit could further reduce disease progression and enhance hepatic regeneration. A retrospective cohort of over 83,000 patients with obesity found semaglutide use associated with a 50-56% lower risk of developing or recurring alcohol use disorder within 12 months^36^.

Safety remains a key consideration. While existing trials in NAFLD and NASH populations have not reported hepatotoxicity, data in decompensated cirrhosis are still limited. A study by Hunyl et al. showed that dual therapy with metformin and GLP-1RA reduced 5-year risk of hepatic decompensation (HR 0.65), especially in women. However, concerns remain about potential interactions, such as GLP-1RA-induced tachycardia or increasing variceal bleeding risk, particularly in patients concurrently using β-blockers^37^. Therefore, prospective studies are needed to better evaluate safety profiles in advanced cirrhosis.

While our study provides compelling evidence supporting the association between GLP-1 receptor agonist (GLP-1RA) use and improved clinical outcomes in patients with cirrhosis, several limitations must be acknowledged.

Firstly, as a retrospective cohort study, our analysis is subject to inherent limitations, including residual confounding and selection bias. Although we performed 1:1 propensity score matching to control for key variables such as age, sex, race, comorbidities (e.g., diabetes, obesity, CKD, hypertension), and lifestyle factors (e.g., alcohol dependence, tobacco use), unmeasured confounders may still have influenced treatment allocation and outcomes. Another shortcoming of our study was that patients who were prescribed GLP-1RAs may have differed systematically from those who were not, beyond what could be adjusted for via propensity matching. We lacked detailed granularity on cirrhosis severity measures such as MELD-Na score, Child-Pugh class, presence of varices, or hepatic venous pressure gradient (HVPG). Our dataset also did not include laboratory markers such as liver enzymes, bilirubin, albumin, or INR, nor imaging or histological findings. These parameters would have allowed for a more nuanced assessment of liver disease progression and fibrosis regression.

We also defined GLP-1RA exposure based on prescription or fill data, but could not verify adherence or duration. Prior studies show dose- and time-dependent differences in outcomes, which we could not explore in depth. The Absence of cause-specific mortality data was also a limitation. Our primary outcome of all-cause mortality does not distinguish between liver-related and non-liver-related deaths. The systemic benefits of GLP-1RAs may influence outcomes beyond hepatic mechanisms.

Another limitation is in respect to generalisability**,** our findings may not apply to patients with advanced decompensated cirrhosis, severe portal hypertension, or cirrhosis without T2DM. Most GLP-1RA users in our cohort were likely in compensated or early decompensated stages. Of note is also that although alcohol dependence was adjusted for, we lacked data on quantity, duration of use, abstinence, or participation in rehabilitation programs, which may confound observed associations.

These limitations underscore the need for prospective, randomized controlled trials in cirrhotic populations to validate our findings and further assess the efficacy and safety of GLP-1RAs in advanced liver disease.

## CONCLUSION

In this large retrospective cohort study, GLP-1 receptor agonist use was associated with significantly reduced risks of all-cause mortality, hepatic encephalopathy, hepatorenal syndrome, and portal hypertensive bleeding in patients with cirrhosis, particularly those with coexisting type 2 diabetes mellitus. These findings support the growing body of evidence suggesting that GLP-1RAs may exert protective effects beyond glycemic control, influencing key pathways in hepatic, renal, and neurologic function relevant to cirrhosis outcomes. Given the burden of cirrhosis-related complications and the limited therapeutic options currently available, GLP-1RAs represent a promising adjunctive therapy in appropriately selected patients. However, further prospective studies and randomized controlled trials are warranted to confirm these associations, establish safety in decompensated stages, and determine the optimal patient populations for treatment.

## Data Availability

Data not available (research data cannot be shared. The data supporting the findings of this study were accessed through the TriNetX research network under license and are not publicly available).
